# Cerium oxide nanoparticles attenuates fibrosis and inflammation in thyroid-associated ophthalmopathy via JNK pathway

**DOI:** 10.3389/fmolb.2025.1580062

**Published:** 2025-07-23

**Authors:** Bo-Wen Wang, Ru Zhu, Ying Jin, Xuan Ouyang, Fa-Gang Jiang, Xing-Hua Wang

**Affiliations:** ^1^Department of Ophthalmology, Tongji Medical College, Union Hospital, Huazhong University of Science and Technology, Wuhan, China; ^2^ Aier Eye Hospital of Wuhan University, Wuhan, China

**Keywords:** thyroid-associated ophthalmopathy, cerium oxide nanoparticles, orbital fibroblasts, JNK phosphorylation, fibrosis, inflammation

## Abstract

**Objective:**

Thyroid-associated ophthalmopathy (TAO) is an autoimmune orbital disorder characterized by pathological alterations including extraocular muscle fibrosis and orbital inflammation. Cerium oxide nanoparticles (CeO2-NPs, CNPs) are gaining popularity in ophthalmology due to their potential antifibrotic and anti-inflammatory properties. This study aims to investigate the inhibitory effects of CNPs on fibrosis and inflammation in TAO orbital fibroblasts (OFs) derived from TAO patients.

**Methods:**

OFs obtained by primary culture of orbital adipose tissue from 8 TAO patients. Probing the safe action concentration of CNPs and Anisomycin using CCK8 and detecting intracellular reactive oxygen species (ROS) generation using ROS Assay kit. Wound-Healing Assay was used to examine the degree of fibrosis of OFs. The expression of Fibronectin, COL1A1, α-Smooth muscle action, Hyaluronan Synthase 2, c-Jun N-terminal Kinase (JNK) and pJNK were detected by the RT-PCR and WB, and Hyaluronic Acid secretion was detected by ELISA. Inflammatory factors Interleukin-6 (IL-6) and Tumor Necrosis Factor-α (TNFα) expression were detected by RT-PCR and ELISA.

**Results:**

CNPs below 100 μg/mL and Anisomycin below 5 μM did not affect OFs proliferation. CNPs inhibit intracellular ROS generation. CNPs inhibit OFs fibrosis and suppress the expression of fibrosis indicators. These antifibrotic effects were mediated by inhibition of JNK phosphorylation, and were reversible by a JNK agonist. Furthermore, CNPs reduce both mRNA levels and secretion of inflammatory factors, IL-6 and TNF-α.

**Conclusion:**

CNPs demonstrated the ability to inhibit fibrosis in TAO OFs by reducing JNK phosphorylation, as well as dose-dependently suppressed ROS generation and inflammatory response in TAO OFs.

## 1 Introduction

Thyroid-associated ophthalmopathy (TAO), also referred to as Graves’ orbitopathy (GO), manifests as an organ-specific autoimmune condition within the context of Graves’ disease (GD) (Zhu et al., 2023). As the predominant extrathyroidal manifestation of Graves’ disease (GD), thyroid-associated ophthalmopathy (TAO) develops in ∼25% of GD patients clinically (Comi et al., 2025). The annual incidence of TAO is 0.54–0.9 (males) versus 2.67–3.3 (females) per 100,000. Mild disease predominates (94%–95% of cases), with moderate/severe forms comprising 5%–6% (Bartalena et al., 2020). The primary ocular manifestations of TAO include exophthalmos, eyelid retraction, and restricted extraocular myopathy (Bartalena et al., 2021). In severe instances, TAO can lead to incomplete eyelid closure, corneal ulcers, optic neuropathy, and potential blindness. Advanced pathological alterations in TAO encompass orbital adipose tissue expansion, fibrosis of the extraocular muscles, and infiltration of inflammatory factors (Diao et al., 2022).

Currently, the etiology of TAO is not fully understood, however, it is believed that the activation of autoimmune T cells and thyroid-stimulating hormone receptor (TSHR) autoantibodies may be significant factors (Shen et al., 2023). Activated immune T and B cells migrate into the periorbital connective tissue, releasing cytokines including IL-1β, IL-6, TNF-α, TGF-β1, and autoantibodies such as IgG, ultimately resulting in connective tissue inflammation, fibrosis, and remodeling (Xu et al., 2022). Oxidative stress also plays a pivotal role in the pathogenesis of TAO. Elevated reactive oxygen species (ROS) levels or diminished antioxidant capacity result in oxidative damage to cellular membranes, lipid peroxidation, and DNA oxidation (Ma et al., 2024).

Treatment varies at different stages of TAO, including orbital decompression surgery, orbital ratiotherapy and medication (Oculoplastic and Orbital Disease Group of Chinese Ophthalmological Society of Chinese Medical Association, 2022). The pathophysiological complexity and interpatient heterogeneity in TAO necessitate personalized management. First-line pharmacotherapy, Mycophenolate mofetil (MMF) plus glucocorticoids (GC) (Längericht et al., 2020) pulse therapy and intravenous teprotumumab (anti-IGF-1R monoclonal antibody) (Markham, 2020), demonstrates robust efficacy when initiated early, modifying disease progression and minimizing complications. Emerging agents [e.g., novel immunosuppressants, somatostatin analogues, and biologic mAbs such as Rituximab (anti-CD20 monoclonal antibody) (Kang et al., 2022), Belimumab (anti-targeting B cell stimulating factor (BAFF) antibody) (Salvi et al., 2019), Tocilizumab (anti-IL-6 antibody) (Boutzios et al., 2023)] show promising efficacy in ongoing trials. However, their cost-effectiveness and safety profiles, particularly long-term adverse events, require rigorous evaluation before clinical translation. Therefore, the research on therapeutic drugs for TAO is still a hot topic at this stage.

Inorganic nanoparticles (NPs) have emerged as versatile platforms for novel therapeutic applications, enabling targeted disease intervention at early pathological stages with efficacy surpassing current treatments (Pelaz et al., 2017). These NPs demonstrate exceptional utility as drug delivery vehicles and dynamic scaffolds that modulate conjugated biomolecule activity (DuRoss et al., 2019). Chen et al. demonstrated that nanoparticles (NPs) synthesized from PLGA-encapsulated TSHR-A subunit and rapamycin (TSHR-A + Rapa NPs) can target dendritic cells (DCs) to modulate immune tolerance, representing a novel potential therapeutic strategy for Graves’ disease (Chen et al., 2025). Cerium oxide nanoparticles (CeO_2_-NPs, CNPs), nanocrystals derived from Cerium, is well known for its antioxidant properties (Casals et al., 2020). Since CNPs can converte between Ce^3+^ and Ce^4+^, it is considered as an antioxidant with auto-regenerative free radical scavenging activity. At present, CNPs have been found to be remarkably effective in eliminate ROS (Li X. et al., 2022), anti-inflammatory (Wei et al., 2021) and anti-fibrosis (Boey et al., 2021), and its neuroprotective effects have numerous applications in ophthalmology, including age-related macular degeneration, retinitis pigmentosa (Maccarone et al., 2020) and acute damage induced by high intensity light exposure (Tisi et al., 2019). The role of CNPs in treating TAO remains unexplored in current literature. Since the pathology of TAO includes oxidative stress, fibrosis of orbital fibroblasts (OFs) and infiltration of inflammatory factors, which is consistent with the role of CNPs, we wished to explore the therapeutic role of CNPs in TAO, and investigate the mechanism.

In this study, we selected OFs derived from orbital tissue of TAO patients, to validate the effect and mechanism of CNPs in TAO extraocular muscle fibrosis and inflammatory infiltration.

## 2 Methods and materials

### 2.1 Materials

Dulbecco’s modified Eagle medium (DMEM), penicillin-streptomycin mixture and 0.25% trypsin-ethylene diamine tetraacetic acid (EDTA) (1х) were purchased from Servicebio (China). Fetal bovine serum (FBS) was purchased from Procell (China). CNPs (544841-5G) was purchased from Sigma-Aldrich (United States). TGF-β (HY-P78168), IL-1β (HY-P7028), Anisomycin (HY-18982) were purchased from MedChemExpress (China).

### 2.2 Samples

The study design and protocol were approved by the Ethics Committee of Huazhong University of Science and Technology Union hospital attached to Tongji University Medical School (UHCT230552), and informed consent was obtained from the patients for the collection of specimens. Orbital adipose tissues were taken from 8 patients, aged between 18 and 65 years old, having no other ocular diseases and major systemic diseases. The clinical and patient information is shown in Table 1. The 7-item clinical activity score (CAS) scheme was used to assess GO activity. When the sum is ≤3/7, TAO is defined as inactive. The severity and clinical activity of GO were graded according to the NOSPECS classification.

**TABLE 1:** The clinical characteristics of donors in this study.

No.	Sex (M/F)	Age	Duration of TAO	CAS	Activity	Previous TAO treatment	Surgery performed
1	F	30	1 year 11 months	0/7	inactive	GCs	Decompression
2	M	43	5 years	0/7	inactive	None	Decompression
3	F	47	1 year	2/7	inactive	GCs	Decompression
4	F	48	11 months	3/7	inactive	GCs	Decompression
5	F	55	1 year 3 months	1/7	inactive	GCs	Decompression
6	F	40	3 years	1/7	inactive	GCs	Decompression
7	M	44	1 year	1/7	inactive	GCs	Decompression
8	F	49	1 year 8 months	2/7	inactive	GCs	Decompression

CAS, clinical activity score; F, female; M, male; y, year; m, month; GCs, glucocorticoids; TAO, thyroid-associated ophthalmopathy.

### 2.3 Primary culture of OFs

Orbital adipose tissue samples were collected and washed three times with phosphate-buffered saline (PBS). Then samples were cut into small pieces of 1–2 mm^3^ and distributed evenly on the bottom of a culture flask. After adding 0.5–1 mL DMEM, the tissue was put into a 37°C cell incubator with 5% CO_2_. When about 80% of the bottom of the flask was occupied, the OFs were digested with 0.25% trypsin for passage. Cells from passages 4–8 were used for subsequent experiments.

### 2.4 Detection of cell proliferation activity

Different concentrations of CNPs and Anisomycin were added to 96-well plates for 2 days. Cell counting kit (CCK)-8 was used to detect the absorbance at 450 nm by using a microplate reader to obtain the action concentration of each drug.

### 2.5 Detection of antioxidative activity

Intracellular ROS generation was assessed using ROS Assay Kit (S0033S, Beyotime, China). DCFH-DA was diluted to 10 μmol/L in serum-free DMEM, added to differently-treated OFs, and avoid the light in 37°C for 20 min. Cells were washed for 3 times before imaging with a laser confocal scanning microscopy (IX51, OLYMPUS, Japan).

### 2.6 Wound-healing assay

Cell migration was detected using an *in vitro* scratch-wound healing assay. 1*10^6^ OFs were seeded on 6-well dishes. When cells reached 90%–100% confluence, different treatments were performed after 12 h FBS-free culture. Linear scratch wounds were made with a 200-μL pipette tip. Images were captured at 0h, 24 h and 48 h under a microscope, and the area of each scratch wound was analysed by ImageJ software.

### 2.7 Enzyme linked immunosorbent assay (ELISA)

Hyaluronic Acid (HA), Interleukin-6 (IL-6) and Tumor Necrosis Factor-α (TNFα) secreted by OFs were detected with corresponding ELISA kits (Elabscience, China). The supernatant was collected after cell processing and assayed according to the manufacturer’s protocols. All experiments were performed in duplicate.

### 2.8 Reverse transcription-polymerase chain reaction (RT-PCR)

Total RNA was extracted from OFs through using the FastPure Cell/Tissue Total RNA Isolation kit (RC101, Vazyme, China) according to the manufacturer’s instructions, and the RNA was reversely transcribed to a complementary DNA (cDNA) by using the HiScript All-in-one RT SuperMix kit (R333-01, Vazyme, China). RT-PCR was performed by using the ChamQ Universal SYBR qPCR Master Mix kit (Q711-02, Vazyme, China). Glyceraldehyde phosphate dehydrogenase (GAPDH) was used as an internal control. Primer sequences were shown in Table 2.

**TABLE 2:** Primer sequence of Fibronectin, COL1A1, α-SMA, HAS2, IL-6, TNFα and GAPDH.

Gene name	Primer sequence
Fibronectin	Forward 5′- TTTTAAGCTGGGTGTACG-3′ Reverse 5′- CAAGTTTGTTGGTGGAGA-3′
COL1A1	Forward 5′- AGGTGTTGTGCGATGACG-3′ Reverse 5′- GGTCGGTGGGTGACTCTG-3′
α-SMA	Forward 5′- CGTGGCTACTCCTTCGTG-3′ Reverse 5′- GTGATGACCTGCCCGTCT-3′
Hyaluronan Synthase 2 (HAS2)	Forward 5′- CTCTTTTGGACTGTATGGTGCC-3′ Reverse 5′- AGGGTAGGTTAGCCTTTTCAC-3′
IL-6	Forward 5′-ACTCACCTCTTCAGAACGAATTG-3′ Reverse 5′-CCATCTTTGGAAGGTTCAGGTTG-3′
TNFα	Forward 5′- TCCAGGCGGTGCTTGTTC-3′ Reverse 5′- TGGCAGGGGCTCTTGATG-3′
GAPDH	Forward 5′- TTAGCACCCCTGGCCAAGG-3′ Reverse 5′- CTTACTCCTTGGAGGCCATG-3′

### 2.9 Western blotting (WB)

After adding Radioimmunoprecipitation assay lysis buffer, the supernatant was collected, and the protein concentration was detected by using a Bicinchoninic Acid (BCA) Protein Assay kit (BL521A, Biosharp, China). Proteins were separated by sodium dodecyl sulfate-polyacrylamide gel electrophoresis, transferred to polyvinylidene fluoride membranes, blocked with 5% Skim milk powder for 1 h at room temperature, and incubated with primary antibodies overnight at 4°C. The primary antibodies used were Fibronectin (1:500, Abmart, China), α-SMA (1:500, Abmart, China), COL1A1 (1:500, Abmart, China), c-Jun N-terminal Kinase (JNK) (1:500, Abmart, China), pJNK (1:500, Abmart, China) and GAPDH (1:10,000, Abclonal, China). After incubated with horseradish peroxidase-labeled goat anti-rabbit secondary antibody (1:2,000, Servicebio, China) for 1 h at 37°C, the protein intensity was detected through using electrochemiluminescence chemiluminescence reagent (ATVK07071, Abbkine, China) and analyzed by using ImageJ software.

### 2.10 Statistical analysis

Graphpad Prism 9.0 and SPSS27.0 software were used for data analysis, and each experimental group was compared with the control group respectively. T-test was applied to analyze the differences between the two groups of data. Shapiro-Wilk Test was used to determine whether the distribution was normal, and Levene’s Test was used to conduct a homogeneity analysis of variance. If the distribution is normal and the variance is homogeneous, an independent sample T-test can be conducted. Mann-Whitney rank sum test was used for non-compliance. The data differences among multiple groups were compared. If the data of each group were normally distributed and the variance was homogeneous, one-way ANOVA was used. As for those which do not meet the requirements, Kruskal–Wallis H test was selected. The above experiments were conducted for more than 3 times, and the results were expressed as mean ± standard deviation. When the P-value was <0.05, the difference was considered statistically significant.

## 3 Results

### 3.1 Action concentration of CNPs and anisomycin on OFs

**FIGURE 1:**
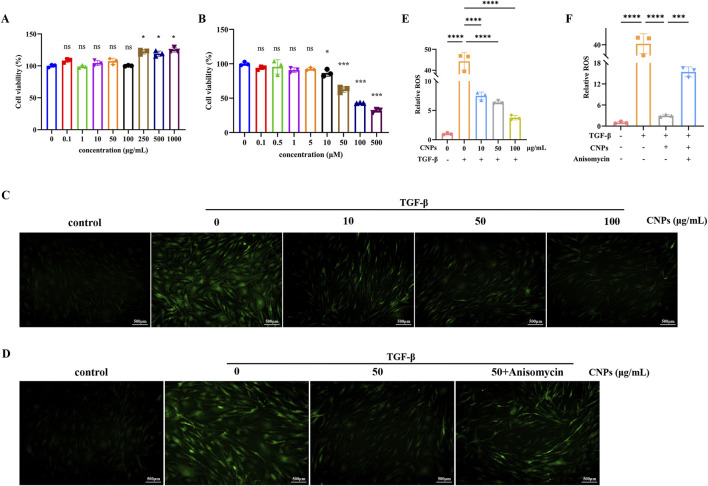
Action concentration of drugs and ROS generation in OFs. **(A,B)** Cell viability measurement using the CCK-8 assay after treatment with CNPs or Anisomycin for 2 days. **(C)** ROS generation of OFs after treatment with 0, 10, 50 and 100 μg/mL CNPs and 5 ng/mL TGF-β. **(D)** ROS generation of OFs in control, TGF-β, CNPs + TGF-β and CNPs + Anisomycin + TGF-β groups. **(E,F)** Fluorescence intensity analysis in Figures **(C,D)**. Data are presented as mean ± SEM (n ≥ 3). ns:*P* > 0.05, *P < 0.05, ***P < 0.001, ****P < 0.0001.

CCK-8 was used to detect the action concentration of CNPs and Anisomycin on OFs. As shown in Figures 1A,B, CNPs ≤100 μg/mL, Anisomycin ≤5 μM did not affect cell activity. Therefore, 10, 50, 100 μg/mL CNPs and 5 μM Anisomycin were respectively chosen for subsequent experiments.

### 3.2 Effect of CNPS on the inhibition of fibrosis and HA secretion in TAO OFs

**FIGURE 2:**
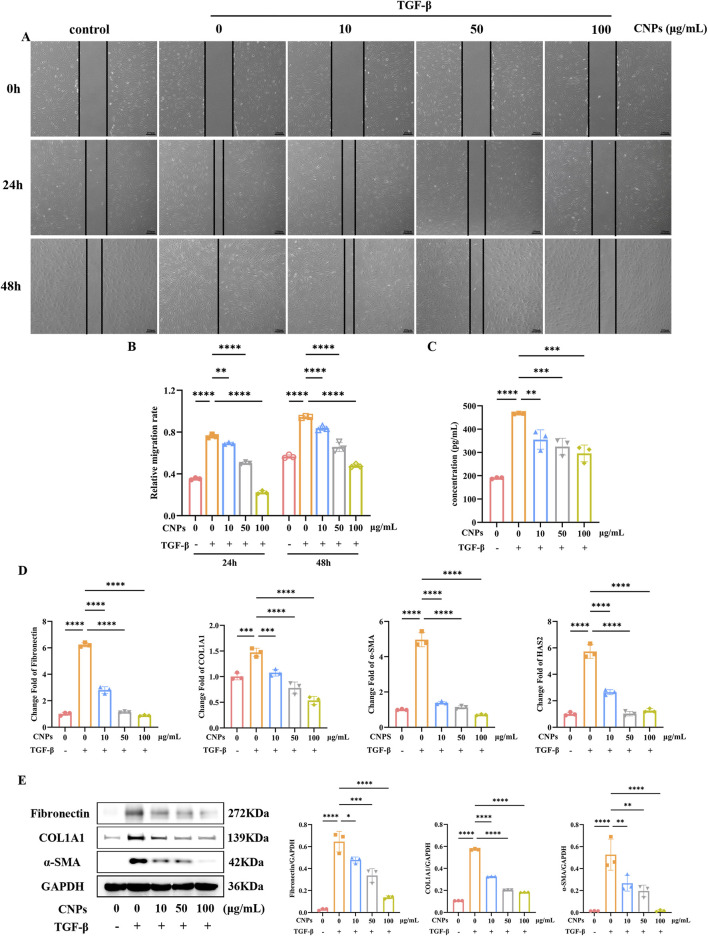
CNPs exert an inhibitory effect on OFs fibrosis. **(A,B)** Wound-Healing assay and cell migration analysis after treatment with 0, 10, 50 and 100 μg/mL CNPs and 5 ng/mL TGF-β, at 24 h and 48 h. **(C)** Secretion of HA after treatment with different concentrations of CNPs. **(D)** mRNA levels of Fibronectin, COL1A1, α-SMA and HAS2 after treatment with CNPs. **(E)** The WB results for Fibronectin, COL1A1 and α-SMA in each group, and the protein levels were quantified and normalized to the level of GAPDH for each sample. Data are presented as mean ± SEM (n ≥ 3). *P < 0.05, **P < 0.01, ***P < 0.001, ****P < 0.0001.

Different concentrations (10, 50, 100 μg/mL) CNPs and 5 ng/mL TGF-β were simultaneously administered to OFs, fibrosis indexes and HA secretion were detected after 2 days. Scratch assays in Figures 2A,B showed significantly higher migration rate of OFs after addition of TGF-β (*P* < 0.05). Meanwhile, addition of CNPs to OFs resulted in a decrease in OFs migration rate (*P* < 0.05), and the gradient of migration rate decreased with increasing CNPs concentration. The mRNA level of fibrosis indicators Fibronectin, COL1A1 and α-SMA in 10, 50 and 100 μg/mL of CNPs groups presented significant reduction, which were 0.45, 0.18 and 0.14-fold; 0.73, 0.53 and 0.36-fold; 0.28, 0.23 and 0.14-fold lower than TGF-β group, respectively (Figure 2D). The WB results exhibited the same trend (Figure 2E). Figure 2C demonstrated the secretion of HA by cells after the addition of different concentrations of CNPs. Similarly, HA secretion was inversely correlated with CNPs concentrations. The mRNA level of HAS2 were 0.46, 0.18 and 0.22-fold, compared with TGF-β group. In summary, CNPs inhibits OFs fibrosis and HA secretion in a dose-dependent trend. Based on this, we selected intermediate concentrations of CNPs (50 μg/mL) for subsequent mechanistic explorations.

### 3.3 JNK pathway mediates CNPs inhibits the process of fibrosis of OFs

Addition of CPNs inhibits intracellular c-Jun N-terminal Kinase (JNK) phosphorylation of OFs. After adding CNPs and inducing fibrosis for 2 days, intracellular fibrosis indicators and pJNK/JNK were examined, which showed a significant reduction in intracellular pJNK/JNK level.

**FIGURE 3:**
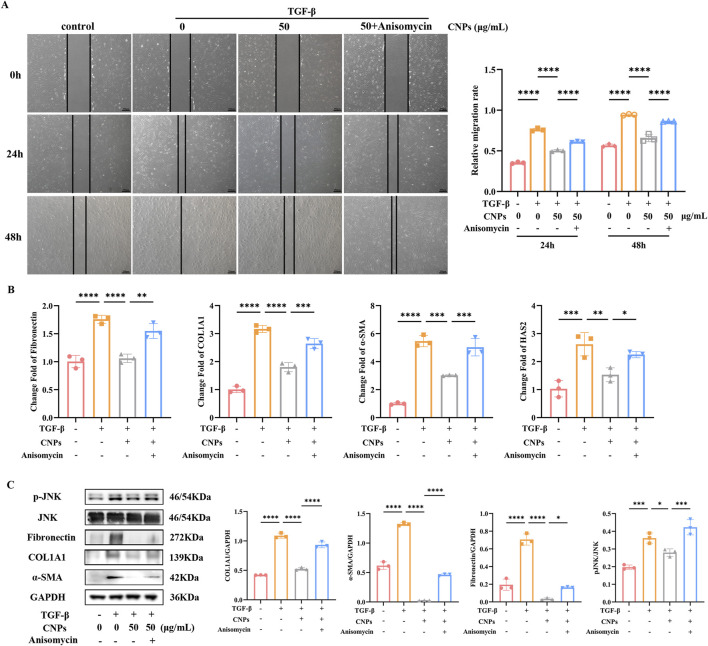
CNPs regulates JNK phosphorylation during OFs fibrosis inhibition. **(A)** Wound-Healing assay and cell migration analysis in control, TGF-β, CNPs + TGF-β and CNPs + Anisomycin + TGF-β groups. **(B)** mRNA levels of Fibronectin, COL1A1, α-SMA and HAS2 in each group. **(C)** The WB results for COL1A1, α-SMA and pJNK/JNK in each group, and the protein levels were quantified and normalized to the level of GAPDH for each sample. Data are presented as mean ± SEM (n ≥ 3). *P < 0.05, **P < 0.01, ***P < 0.001, ****P < 0.0001.

Anisomycin was chosen to verify the inhibition of JNK phosphorylation in CNPs-mediated inhibition of fibrosis in OFs. Wound-Healing Assay showed the migration rate of OFs. Compared to CNPs group, CNPs + Anisomycin group exhibited significant increase, either 24 h or 48 h after fibrosis induction (*P* < 0.05) (Figure 3A). The mRNA expression of Fibronectin, COL1A1, α-SMA and HAS2 in CNPs + Anisomycin group were significantly higher than CNPs group, which were 1.50, 1.47, 1.66 and 1.47-fold, respectively (Figure 3B). Protein level of Fibronectin, COL1A1 and α-SMA in OFs followed the same trend as mRNA. CNPS induced lower JNK phosphorylation than that in TGF-β group and CNPs + Anisomycin, which were 1.29 and 1.51-fold (Figure 3C). These suggest that CNPs inhibits fibrosis of OFs via reducing the phosphorylation of JNK.

### 3.4 Effect of CNPs on the inhibition of ROS generation in TAO OFs

Different concentrations (10, 50, 100 μg/mL) CNPs and 5 ng/mL TGF-β were simultaneously administered to OFs, occurring with 50 μg/mL CNPs + 5 μM Anisomycin group, and intracellular ROS level were observed by microscopy. As shown in Figures 1C,E, compared with induction group, intracellular ROS generation were significant decreased in 10, 50, 100 μg/mL CNPs group in a dose-dependent manner. Furthermore, intracellular ROS level of CNPs + Anisomycin group showed somewhat elevated compared to CNPs group (Figures 1D,F).

### 3.5 Effect of CNPs on the inhibition of secretion of inflammatory factors in TAO OFs

**FIGURE 4:**
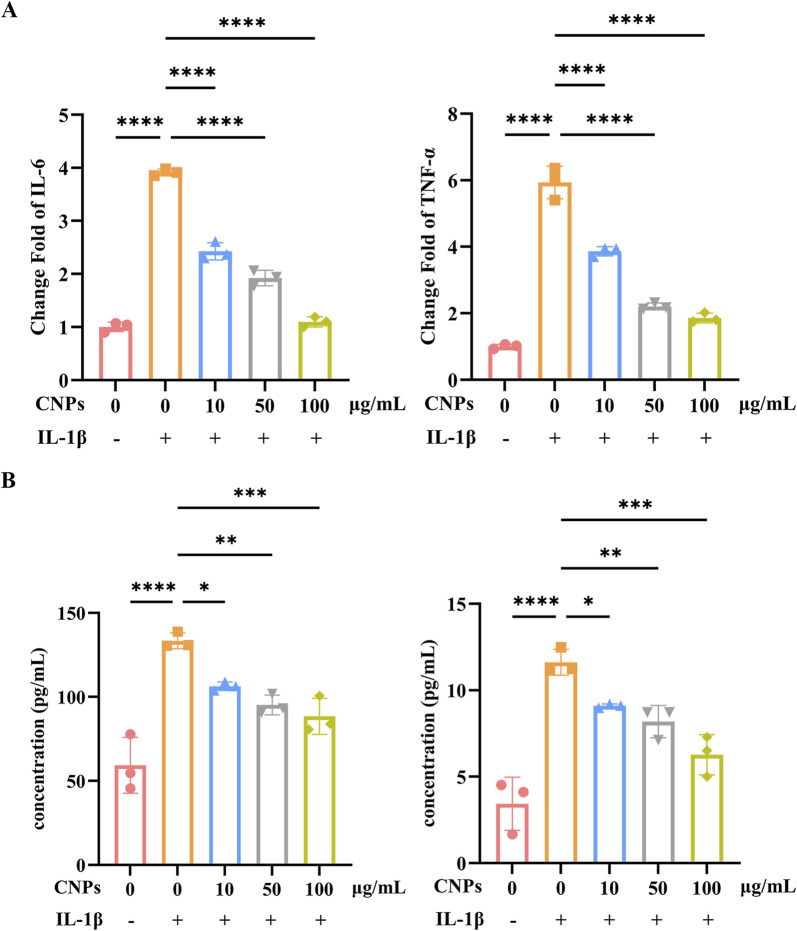
CNPs exert an inhibitory effect on OFs inflammation. **(A)** mRNA levels of IL-6 and TNF-α after treatment with 0, 10, 50 and 100 μg/mL CNPs and 5 ng/mL IL-1β. **(B)** Secretion of IL-6 and TNF-α after treatment with different concentrations of CNPs. Data are presented as mean ± SEM (n ≥ 3). *P < 0.05, **P < 0.01, ***P < 0.001, ****P < 0.0001.

Different concentrations (10, 50, 100 μg/mL) CNPs and 5 ng/mL IL-1β were simultaneously administered to OFs, the mRNA level and secretion of inflammatory factors, IL-6 and TNFα, were detected after 24 h. The expression of IL-6 and TNFα were significantly downregulated, which were 0.62, 0.49 and 0.28-fold; 0.65, 2.21 and 0.31-fold, respectively, as determined using RT-PCR (Figure 4A). Trends in IL-6 and TNFα secreted by OFs are identical, which detected by ELISA, and presented 0.80, 0.72 and 0.67-fold; 0.78, 0.71 and 0.54-fold, lower than induction group (Figure 4B). Both mRNA level and secretion of IL-6 and TNFα decrease with increasing CNPs concentration.

## 4 Discussion

Thyroid-associated ophthalmopathy (TAO) is a refractory orbital disease characterized by specific immune reactivity and pathological remodeling of periocular tissue (Li Z. et al., 2022). Oxidative stress is one of the most important mechanisms underlying the pathogenesis of TAO, leading to elevated ROS, which could result in fibrosis and inflammation. Extraocular muscle fibrosis and inflammatory reaction are mean pathologic changes in TAO. Acute TAO is driven by Th1-mediated inflammation (Antonelli et al., 2014), chronic inactive TAO is characterized by progressive fibrosis. Inflammatory infiltration and accumulation of glycosaminoglycans (GAG) lead to edematous-infiltrative changes in periocular tissues (Łacheta et al., 2019) while TGF-β stimulated differentiation of OFs into myofibroblasts (Hao et al., 2020), which in turn leads to ocular dyskinesia, restrictive extraocular myopathy, and other clinical symptoms in patients. Currently, conventional treatments for TAO include glucocorticoids, immunosuppressants, orbital radiotherapy (Zhang X. et al., 2023), and strabismus correction, but the available treatments have many side effects, such as cushingoid features, elevated blood pressure, weight gain, muscle pain and osteoporosis (Zang et al., 2011). In the active phase, patients feel red and painful in eyes because of vasodilatation and inflammatory cell infiltration caused by active inflammation, and respond well to immunosuppressive therapy. In the inactive phase, with fibrosis taking place, patients show painless motility deficit in eyes, and immunosuppressive therapy is useless (Lin et al., 2021). Surgical interventions provide only symptomatic relief, with many patients experiencing poor prognoses. In recent years, targeted therapies for TAO have developed rapidly. Monoclonal antibody drugs, for example, Teprotumumab (Markham, 2020) and Rituximab (Kang et al., 2022), have emerged as novel therapies for TAO. Teprotumumab, a monoclonal antibody that inhibits IGF-1R, is the first disease-modifying therapy approved in the United States for the treatment of TAO in the United States (Nie and Lamb, 2022). Rituximab, a monoclonal antibody specifically binding CD20, was the first biologic therapies applied to the treatment of active TAO (Genere and Stan, 2019). Although these medications are more effective than traditional treatments, their prohibitive cost limits accessibility. Consequently, the search for a safe, cost-effective, and highly effective TAO therapeutic agent is the current focus of scientific research.

CNPs are cerium oxide particles with a diameter of less than 100 nm, which exhibit strong antioxidant capacity attributed to the unique electronic orbital structure of cerium atoms, which enables the interconversion between Ce^4+^ and Ce^3+^ states (Singh et al., 2020). In recent years, CNPs have garnered significant research interest within the medical field, particularly for their neuroprotective and antioxidant properties mediated through the reduction of ROS generation. They also have numerous applications in ophthalmology. The protective role of CNPs in retinal disease in rats (Wong et al., 2013) and mice (Cai et al., 2016) has been demonstrated. Chen et al. found that intravitreal injection of CNPs prior to light exposure effectively protects photoreceptor health and retinal function in albino rats (Chen et al., 2006). CNPs also prevented light-induced degradation of RPE and accumulation of lipofuscin (Tisi et al., 2019). In addition, the investigators tested the lens epithelial cell toxicity of CNPs *in vitro* (Pierscionek et al., 2012) to confirm that CNPs are also a potential cataract treatment strategy. At the same time, the anti-fibrotic and anti-inflammatory studies of CNPs are accumulating. CNPs exert an inhibitory effect on hepatic fibrosis in mice by reducing ROS production (Godugu et al., 2023) and significantly attenuate the inflammatory response in rats with CCL4-induced liver injury (Oró et al., 2016). Therefore, we assessed the suppression effect of CNPs on TAO OFs fibrosis and inflammation, and explored the mechanism.

In our study, we firstly verified the safe concentration of CNPs on OFs. We demonstrated that CNPs inhibited the fibrotic process and inflammatory response of TAO OFs in a dose-dependent manner. Meanwhile, we found a gradient decrease in intracellular ROS generation with increasing CNPs concentration.

In one study, CNPs were found to downregulate the p38/JNK signalling pathway after intravitreal injection and to inhibit neovascularisation in vldlr−/− mice (Cai et al., 2014). Li et al. further reported that CNPs downregulate JNK phosphorylation in human skin fibroblasts after UVA irradiation to against UVA radiation-induced photoaging (Li et al., 2019). These suggested that CNPs inhibit JNK phosphorylation. JNK, a primary member of the Mitogen-Activated Protein Kinase (MAPK) family, regulates diverse biological processes including cell proliferation, differentiation, cytoskeleton construction, and apoptosis (de Los Reyes Corrales et al., 2021). Evidence implicates resistin promotes cardiac fibroblast to myofibroblast transformation by activating JNK pathway (Singh et al., 2021). Zhang et al. reported that targeting GPR65 alleviates hepatic inflammation and fibrosis by suppressing the JNK pathways (Zhang K. et al., 2023). Otherwise, Astragalus polysaccharide alleviated alcoholic-induced hepatic fibrosis by inhibiting TLR4/JNK/NF-κB/MyD88 pathway (Sun et al., 2023). These suggested that attenuating JNK phosphorylation inhibits fibrosis.

Accordingly, we assessed the degree of cellular JNK phosphorylation upon the addition of 50 μg/mL CNPs when fibrosis was induced and found that the level of CNPs significantly reduced phosphorylated JNK compared to untreated controls. Co-treatment with the JNK agonist Anisomycin revealed an increase in the cellular JNK phosphorylation level and a significant rebound in intracellular ROS generation and fibrosis compared to the addition of CNPs alone. Oxidative stress is associated with the JNK pathway (Gehi et al., 2022). Study showed that blocking JNK pathway alleviates alcoholic liver fibrosis by inhibiting oxidative stress (Liu et al., 2022). *In vitro* JNK inhibitor reduces oxidative stress in SH-SY5Y cells (Rehfeldt et al., 2021). Collectively the above experimental results suggest that CNPs are greatly likely to alleviate oxidative stress by attenuating JNK phosphorylation, thereby inhibiting fibrosis of OFs. Simultaneously, suppress OFs inflammatory response in a dose-dependent manner.

Nevertheless, our study has certain shortcomings. First, there is no more recognized and stable animal model of TAO for validation in *in vivo* experiments. Second, our investigation provided a preliminary exploration of the mechanisms by which CNPs inhibit fibrosis in OFs. Third, the sample size of eight patients was relatively small. Given CNPs’ demonstrated efficacy in treating other ophthalmic diseases, we believe that the results of this study are valuable for the use of CNPs in the treatment of TAO.

## 5 Conclusion

The present study confirmed that CNPs inhibited TAO OFs fibrosis by attenuating JNK phosphorylation, while dose-dependently suppressing TAO OFs inflammatory response and oxidative stress. These findings establish a theoretical foundation for utilizing CNPs, a novel, cost-effective, and safer therapeutic agent, in the treatment of TAO extraocular muscle fibrosis and inflammation.

## Data Availability

The original contributions presented in the study are included in the article/supplementary material, further inquiries can be directed to the corresponding authors.
